# Pharmacovigilance for Vaccines Used in Pregnancy: A Gap Analysis From Uganda

**DOI:** 10.1097/INF.0000000000004705

**Published:** 2025-02-14

**Authors:** Victoria Nambasa, Anthony Ssebagereka, Agnes Ssali, Ritah Namugumya, Phionah Nalubega, Albert Figueras, Dan Kajungu, Birgit Koch, Kirsty Le Doare, Beate Kampmann

**Affiliations:** * African Union Development Agency – New Partnership for Development (AUDA-NEPAD), Johannesburg, South Africa; † Department of Hospital Pharmacy, Erasmus University Medical Center, Rotterdam, Netherlands; ‡ Makerere University School of Public Health, Kampala, Uganda; § Makerere University – John Hopkins University Research Collaboration, Kampala, Uganda; ¶ Medical Research Council/ Uganda Virus Research Institute & LSHTM Uganda Research Unit. Entebbe, Uganda; ‖ Independent Researcher / Pharmacovigilance Consultant, Barcelona, Spain; ** Makerere University Centre for Health and Population Research [MUCHAP], Kampala, Uganda; †† Stellenbosch University, Stellenbosch, South Africa; ‡‡ Centre for Neonatal and Paediatric Infection, St George’s, University of London, London United Kingdom; §§ Charité Centre for Global Health, Charité Universitätsmedizin, Berlin, Germany; ¶¶ Faculty of Infectious & Tropical Diseases, London School of Hygiene & Tropical Medicine, London, UK

**Keywords:** maternal vaccines, LMICs, vaccine safety, pharmacovigilance, electronic medical records

## Abstract

**Background:**

Despite the effectiveness of maternal vaccines, low-and middle-income countries grapple with inadequate safety monitoring systems. Robust safety surveillance is crucial to increasing vaccine confidence and timely identifying any potential safety signal that could put pregnant women and children at risk following vaccination. This study assessed the pharmacovigilance systems for vaccines used in pregnancy in Uganda.

**Methods:**

A qualitative study involving thirteen key informant interviews and eight focus group discussions (FGD) among key stakeholders. Purposive sampling was used to select study participants. Data analysis was done using Miles and Huberman’s matrices approach and conducted in Atlas-ti software.

**Findings:**

A passive system involving multi-stakeholders at various levels of the healthcare structure existed but was inadequate for monitoring Adverse Events Following Maternal Immunization (AEFMI). Existence of parallel reporting systems for vaccines was noted. Heavy workload, lack of feedback, inadequate knowledge to recognize and report AEFMIs, and logistical challenges impeding reporting and follow-up were among the barriers to reporting. Electronic Medical Records (EMR), though underutilized for safety surveillance, offer promising potential.

**Conclusion:**

To address the specific needs of maternal vaccination, the pharmacovigilance system in Uganda needs improvement. A multi-pronged approach, including policy coherence, embracing active surveillance, and leveraging existing birth outcome surveillance and EMR, is essential. Harnessing healthcare provider knowledge and advisory committee capacity in causality assessment is also necessary. The study findings can inform priority interventions to enhance pharmacovigilance for existing and new maternal vaccines.

## Background

Pregnant women and infants are susceptible to numerous vaccine-preventable diseases, particularly in resource-constrained settings, where nearly 2.3 million infants die annually, often due to preventable infections [1, 2]. Vaccination during pregnancy provides protection to pregnant woman and her infant through antibodies that can pass through the placenta to stimulate the infant’s immune system, thereby providing an effective defense against potential complications prior to the infant’s vaccination taking effect [1–3]. This preventive strategy offers a promising path to saving lives and promoting healthy beginnings for infants and mothers worldwide [4–6].

The World Health Organization (WHO) recommends several vaccines for pregnancy, including vaccines against maternal and neonatal tetanus, influenza, and infant pertussis [7–11]. The tetanus vaccine has been routinely used in low and middle-income countries (LMICs), and its safety profile has been demonstrated despite limited monitoring systems for antenatal vaccination [2, 12–14]. However, the safety of other vaccines used, for example, in disease outbreaks in LMICs, has not been extensively studied. The anticipated deployment of new vaccines targeting diseases such as respiratory syncytial virus (RSV) and Group B streptococcus (GBS) is imminent to reduce newborn morbidity and mortality. Some new vaccines employ novel technologies underpinning the need for strengthened safety surveillance systems [2, 15–19]. Currently, LMIC safety surveillance systems are predominantly passive, thus presenting challenges in monitoring and analyzing adverse events following maternal immunization (AEFMI) [20]. Passive systems suffer from several limitations, including underreporting, varying data quality, lack of a denominator data to obtain incidence rates, and lack of background information on medical events [15, 21, 22]. Systems that are integrated into current safety reporting practices and can systematically track and monitor the outcomes of pregnant women and their infants over time are necessary for both new vaccines and those used in pandemic or disease outbreaks [15]. Electronic medical records, custom software platforms (e.g. District Health Information System) and research networks (Health and Demographic Surveillance systems) are available in resource-limited settings with potential to collect data and tracking the progress of pregnant women and their infants during the perinatal and postnatal periods. However, there has been limited utility of these systems for post-marketing surveillance of vaccines administered to pregnant women, underscoring the need to adopt them for the surveillance of vaccine safety in pregnancy [21–23].

Uganda recommends that pregnant women receive two doses of the tetanus-diphtheria vaccine, with a maximum of five doses to women in childbearing years (ages 15-45)[24]. During the COVID-19 pandemic, Ugandan pregnant women were also offered COVID-19 vaccines despite a lack of safety data in pregnancy. In addition, vaccines not generally recommended during pregnancy have been occasionally administered during disease outbreaks and mass vaccination campaigns [25, 26]. Uganda operates a pharmacovigilance system [27], however structures, functions and people constraints mean that the current system requires strengthening to conduct safety surveillance from maternal immunization. As more maternal vaccines are offered in pregnancy in Uganda, building a robust Pharmacovigilance (PV) system that can capture events related to maternal immunization is essential. Such a system must be equipped to monitor known AEFMIs and potential new and rare events.

This study aimed to map stakeholders, identify gaps in existing pharmacovigilance systems, and assess the potential for utilizing electronic medical records and registries to monitor AEFMI in Uganda.

## Methods

### Study Design

This study employed a qualitative approach, utilizing key informant interviews and focus group discussions. The interview guide (Supplementary material) was structured to assess the core components of a comprehensive Pharmacovigilance (PV) framework which incorporates activities and resources at the health facility, national, and international levels and foster collaboration among various partners and organizations that contribute to ensuring medicine safety [28]. The framework domains include the people (adverse event reporters and evaluators), functions (data collection, data evaluation, and data utilization for decision-making) and structures (Ministry of Health, regulatory agency/pharmacovigilance center, industry, and advisory committees, among others). The interview questions explored two key areas: general PV structures, processes, and those tailored explicitly for maternal vaccines. Prior to implementation, the interview guide underwent testing and refinement through role-play exercises with the research assistants responsible for data collection. The study adopted a relationship-based approach to map stakeholders, guided by the Pharmacovigilance framework.

### Study Sites, study population and sample distribution

In the context of the pharmacovigilance (PV) framework, various stakeholders were actively engaged in the study, including policymakers at the national level (Ministry of Health (MOH), WHO country office), the drug regulatory agency/pharmacovigilance center (National Drug Authority (NDA)), and public health programs (immunization program, Maternal and Child Health), as well as implementers (health facilities providing antenatal care (ANC)). A purposive and representative sampling technique was employed to select participants from these identified stakeholders, ensuring the collection of insights pertinent to our research objectives.

Uganda’s public health care system operates on a decentralized model, with lower-level facilities including Health Centre IIs, Health Centre IIIs and Health Centre IVs being managed by the districts while the Ministry of Health oversees the hospitals [29]. We employed purposive sampling to select five health facilities across three geographical regions: Eastern, Western, and Central Uganda. The sample frame prioritized regional hospitals, district hospitals, and Health Centre IV/III, which were easily accessible and active in reporting adverse events.

In focus group discussions, we engaged healthcare workers involved in delivering maternal vaccinations at the selected facilities. We also conducted interviews with representatives from research institutions, i.e. Infectious Diseases Institute [30], Baylor Uganda [31], Makerere University -John Hopkins University Research Collaboration [MU-JHU] [32], and the Medical Research Council/Uganda Virus Research Institute and London School of Hygiene and Tropical Medicine Uganda Research Unit [33]. These interviews focused on their involvement in pharmacovigilance for clients receiving routine health care in their research studies and provided insights into the use of EMR and drug registries.

### Eligibility

The study included individuals who met the criteria for knowledge in vaccination and/or pharmacovigilance and provided written or oral (those who were not physically available for interviews) consent to participate.

### Data collection

#### Key Informant Interviews

We conducted thirteen interviews with diverse representation using a Semi-structured interview guide (see Guide, Supplemental Digital Content 1) led by skilled investigators in qualitative health research (ELO, RK, VN,) and supported by a research assistant. Interviews were spread over a period of 3 months (April-June 2023) to allow for the scheduling of appointments with participants. Ten interviews were conducted face–to–face in their offices and health facilities, while three of the participants (one each from MOH, WHO and NDA) were interviewed virtually. All KIIs were conducted in English. Interviews took at least 90 minutes and were audio-recorded.

#### Focus Group Discussions

After obtaining administrative clearance from facilities, we conducted five focus group discussions among healthcare providers involved in ANC, immunization services and Pharmacovigilance in selected health facilities. The study targeted 40 participants to be enrolled in the FGDs with eight participants per group. However, one of the FGDs had seven participants. A skilled social worker with PV knowledge (RK, DBN, RNK) supported by a research assistant led discussions. We first introduced the study to all relevant staff at these facilities, and those interested in participating were then selected. The research team continuously assessed for and later confirmed that saturation was achieved with the declared number of interviews.

### Data analysis

We adopted Miles and Huberman’s[1994] [34] matrices for qualitative data analysis. The first step was transcribing the data, followed by open coding using inductive and deductive coding to identify emerging themes and subthemes. The data analysis involved a combination of inductive and deductive coding applications using a pre-developed codebook with a priori codes. Codes were changed, and emerging codes were added through the inter-coder reliability process, only if deemed necessary by the whole coding team. The codebook was finalized and applied to all the transcripts, before final analysis, using a consistent set of codes and a uniform understanding of how to apply them. A coder reliability check was performed on 10% of coded transcripts selected randomly throughout the coding process. Later, we uploaded the transcripts to ATLAS.ti version 9 to generate a query report. [35]. Once all the data was coded, the team synthesized the data under parent codes to identify and analyze relationships between differently coded data. The codes were entered into a master Excel sheet, and all text relating to the code was assigned to the respective code.

## Results

### Characteristics of Participants

We conducted five (5) focus group discussions (FGDs) with healthcare workers from selected health facilities and 13 key informant interviews (KIIs) representing various stakeholders, as detailed in Table, Supplemental Digital Content 2. Four of the FGDs comprised eight participants, while one group had seven participants. All the thirty-nine participants in FGD were female with a mean age of 41.8 years [median= 43 [range=26-59]].

Our key findings are presented with detailed excerpts from the respondents as Table, Supplemental Digital Content 3.

### Product Scope used in maternal Vaccination

Tetanus Toxoid Td [MV1+] and Tetanus—diphtheria [Td] were routinely administered, while COVID-19 vaccines were given to pregnant women during the pandemic. Additionally, vaccines for Hepatitis B, Cholera, Meningitis, and Yellow fever were also used during vaccination campaigns and disease outbreaks, including during pregnancy.

### Stakeholders involved in vaccine Pharmacovigilance

Stakeholders at various levels of the healthcare system had distinct roles within the PV ecosystem as detailed in Table, Supplemental Digital Content 4, and Figure, Supplemental Digital Content 5. However, at the time of this study there was no dedicated program for pharmacovigilance of maternal immunization. The World Health Organization (WHO) provided international policy guidance for vaccination, while the coordination of pharmacovigilance activities of medicines and vaccines in collaboration with other stakeholders was the mandate of the National Drug Authority (NDA). The Ministry of Health (MOH) and the Uganda National Expanded Programme on Immunization [UNEPI] are instrumental in shaping policies and developing guidelines for the use of vaccines and monitoring for quality and safety along the distribution chain. The National Adverse Events Following Immunisation (AEFI) Committee, established by the MOH, was responsible for providing technical guidance on the classification and causal assessment of serious AEFIs. The committee’s role included evaluating the relationship between vaccines and reported adverse events and providing specific recommendations to enhance vaccine safety protocols.

*“I would not say we have a special arm dedicated to pregnant women, but generally, we work with UNEPI and WHO to monitor all vaccines used. We have a National AEFI expert committee that periodically reviews and classifies serious AEFI and gives recommendations*…*”* [**KII_NDA**]

Additionally, non-governmental organizations, implementing partners, and research institutions supported pharmacovigilance-related activities in data collection or capacity building for healthcare workers.

At the district level, healthcare providers and district surveillance officers were the primary actors responsible for implementing pharmacovigilance activities. Regional referral hospitals, serving as regional pharmacovigilance centers, worked closely with the NDA to oversee and coordinate the reporting and monitoring of adverse drug events within their regions.

### Methods for monitoring safety, tools, and information flow

Generally, the PV system was set up as a passive reporting system. There was no specific official approach for monitoring the use of vaccines in pregnant women except through the general PV system used for all health products. AEFI, if it occurred in pregnant women, was reported using tools provided by the NDA. Nevertheless, there were some efforts to implement active surveillance for other products, like the Dolutegravir-based regimen used to manage HIV and COVID-19 vaccines in sentinel sites

*“We don’t have a special system for pregnant women. We rely on the surveillance system for all other medicines and vaccines. Therefore, if it can work for the children’s vaccines, it can also work for pregnant women. So, it’s the same system by the health workers interacting with these women and the same reporting system*.*”* [**KII_MOH**]

*“Before the pandemic, the surveillance was ordinary passive surveillance, which we mainly did to get childhood vaccine-related reactions. Then, after the pandemic, the USSD code was instituted so patients could report. That was the first instance where surveillance was put in the hands of the patients, and they could actively send us reports of what had happened after receiving the vaccine*.**” [KII_NDA]**

The number of reported AEFI in pregnant women was very low. According to respondents from the National Drug Authority, the database contained only six reports related to maternal vaccines used routinely (Tetanus Toxoid) out of 16,000 reports collected between 2007 and the time of data collection (2023), while 95 reports of tetanus toxoid-containing vaccines were reported in children below 2 years old, which is a clear example of underreporting. This disparity may be attributed to a lack of concern among healthcare providers and women regarding expected or common vaccine side effects, leading to underreporting.

*“With vaccines, I don’t know if it’s because many health workers tell the people receiving vaccines that all these side effects are normal. When they are sending them home, they tell them that “you will get fever, of course you will get injection site pain, and of course this will happen “. So vaccine recipients rarely think of reporting. They are like “we thought that it was normal for someone to get an abscess on the shoulder*.*” [KII_NDA]*

The study found no specific data collection tools to handle AEFMI other than tools used within the general PV system for vaccines. Data collection tools included paper-based ADR/AEFI reporting forms and an electronic reporting form for healthcare workers. To enhance self-reporting, additional platforms such as a toll-free hotline, a USSD code, the Med Safety mobile app and WhatsApp were made available to both healthcare workers and the public [36, 37]. Any identified AEFI was first recorded in the vaccination register at the health facilities before being transferred to the NDA reporting tools. The NDA utilized VigiFlow, a web-based WHO platform, to manage this data.

*“…I think we need to look at the tools, and one of the variables we should consider is whether the mother has been vaccinated during pregnancy; any side effects should be documented. We need to review the existing tools to capture needs for mothers receiving vaccines…”* [**KII_WHO**]

*“… But I would not say that someone would take a particular interest and sit down to look at specifically pregnant mothers. Occasionally, there will be reports of miscarriages, but establishing causality for those reports is very difficult because sometimes whatever the mother is being treated for, is also a risk factor for a miscarriage. Then tracing back, to be fair, we have not really done that. I would not say we do specific safety digging for pregnancy*. **[KII_NDA]**

*“Because it’s even rare. Now this goes back to the report quality, it’s rare for the reporter to specify that this was a pregnant mother or this was a lactating mother’*. ***[KII_NDA]***

The District Health Information System [DHIS2], a web-based platform for district health reporting software, was also employed to report AEFIs alongside other health data in public health facilities. Immunization information was initially recorded in a vaccination register before being transferred into the DHIS2.

Some health facilities involved in collecting data for research reported using an Open Data Kit [ODK] - based data collection system, an open-source tool for collecting data using Android mobile devices. For example, four major hospitals in Kampala were collecting hospital-based birth defects using this tool.

### Electronic Medical Records and registries and their interface with the National Drug Safety Reporting System

An electronic medical record system [referred to as UgandaEMR developed by the MoH as a point-of-care system to aid patient management, clinical documentation, and reporting at the point of service within a health facility service was available. This system was reported to be in use across more than 360 public and private health facilities.[38]. The system was mainly implemented in HIV clinics; however, plans were underway to incorporate all healthcare services beyond HIV. Importantly, the system was not linked to the NDA safety reporting system.

EMRs and registries were also found in research institutions like Baylor and IDI, which captured patient clinic data and information on vaccines and drugs administered to clients. However, their systems were primarily set up for HIV/AIDS care, and data on pregnancy outcomes were not shared with the NDA or the MOH.

“…*we have our electronic patient clinic management tool where we complete all the routine clinic data*. we do not currently report to the ministry or National Drug Authority on the outcomes of our mothers. We are only reporting to the Antiretroviral Pregnancy Registry…It is a USA-funded database.” [**KII_IDI**]

### Challenges faced with reporting adverse drug/vaccine events

Stakeholders identified several logistical and operational challenges around reporting adverse drug events, including those of vaccines as presented in Table, Supplemental Digital Content 6.

## Discussion

Our study demonstrates that the existing system for monitoring vaccines administered to pregnant women in Uganda needs improvement to support the monitoring and detection of relevant AEFMI. This is the first study in Uganda to provide an in-depth analysis of how maternal vaccines are currently monitored within the broader context of surveillance structures used for all health products.

One significant finding was the absence of a targeted strategy focusing on vaccines used in maternal immunization. The reporting system was generic, without recognizing the uniqueness that PV for vaccines used in pregnancy may present. Relying on passive surveillance as the primary method of data collection to monitor vaccines used in maternal immunization poses challenges, such as underreporting of adverse events and health outcomes in pregnant women.

While tetanus vaccines have been routinely used for a considerably long time, the introduction of other vaccines administered to pregnant women has not considered the need for an appropriate framework for monitoring potential adverse events in this population. Additionally, the reporting system is fragmented with many parallel PV systems that are not integrated into a central information hub, thus limiting the ability to detect and respond to safety signals effectively.

However, a “silver lining” emerged; stakeholders across the healthcare system were working to ensure vaccine safety and appropriate use. Harnessing collaboration among all stakeholders through discussions centered on an integrated approach to maternal vaccine safety monitoring is crucial. This collective effort aligns with previous studies conducted in LMICs [16, 17, 27], which have emphasized the importance of leveraging such collaborations to strengthen and build more robust PV systems for maternal vaccines.

The study revealed that a minimal proportion, less than 1%, of AEFI reports from the NDA pertained to pregnant women receiving routine maternal vaccines, such as tetanus, despite the administration of other vaccines during mass vaccinations. This observation may be attributed to the perception that the side effects experienced are mild and within the range of normalcy, thus not warranting formal reporting. In discussions with healthcare workers, we found that during educational sessions, women are advised that the side effects they may experience are mild and expected, potentially contributing to underreporting. A systematic review indicated that a lack of knowledge regarding adverse drug reaction (ADR) reporting—where healthcare professionals perceive that only serious ADRs necessitate reporting—significantly contributes to the underreporting of ADRs[39]. Moreover, it suggests a potential inadequacy in the current system’s capacity to discern adverse events in pregnant women beyond the readily identifiable reactogenic effects of vaccines.

Healthcare workers encountered several challenges when it came to reporting, such as [i] lack of feedback, which discouraged further participation; [ii] heavy workload and competing demands, leaving little time for AEFI reporting; [iii] inadequate knowledge to identify and report AEFIs; [iv] incomplete medical information, which made causality assessments difficult; and [v] logistical challenges, such as limited internet access, which hinder both reporting and follow-up with clients. Similar challenges have been reported in studies conducted globally, including in low- and middle-income countries [40–42].

While Inman’s “seven deadly sins” described underreporting decades ago [43], our study identified additional barriers. The lack of feedback and incentives diminish motivation, while logistical hurdles like poor internet access create practical barriers. Difficulties accessing the internet could be overcome by providing healthcare facilities with free internet hotspots, thus improving internet access and facilitating reporting of these events. Finally, monetary incentives to report were a frequently cited topic; however, previous research shows that this leads to an unsustainable dependence on payment [44].

Our findings indicate that healthcare professional education is essential. Healthcare professionals need to know the importance of safety reporting and recognize that it is part of their professional duties, not an optional task for which remuneration should be expected. Furthermore, failing to identify an adverse reaction can be a form of misdiagnosis, and reporting any suspected adverse outcome to a vaccine or medicine is integral to good healthcare practice [45].

### Facing the future

As proposed in Figure, Supplemental Digital Content 7, a multi-pronged approach is necessary to strengthen the pharmacovigilance of maternal vaccines in Uganda. Establishing a dedicated policy and agenda for monitoring AEFMI is needed to foster collaboration among multiple stakeholders. Active Pharmacovigilance offers a promising complement for passive surveillance, especially for monitoring new vaccines, targeted campaigns, and specific severe reactions/adverse events of special interest [15].

While the efficiency of active monitoring is well established, resource constraints within the Ugandan context must be carefully considered. A practical solution is building upon existing, less resource-intensive efforts like the ODK-based birth defect surveillance system while customizing to link vaccination and infant data in sentinel sites. This system could be readily adapted through brief training for healthcare professionals attending deliveries, [midwives, and OB-GYNs on the safety monitoring system, its importance, and reporting procedures [46, 47]. The existing DHIS platform and the health and demographic surveillance available in the country could be explored for active surveillance of AESI related to maternal vaccination [23]. Provision linking infant data with maternal records is essential to monitor maternal and infant outcomes. This is important because pregnancy vaccination impacts maternal safety and infant health; hence, postnatal follow-up needs [15]. Therefore, tools that link the antenatal and postnatal care records need to be explored. Additionally, it is essential to reiterate the basic principle of pharmacovigilance: the possibility of an adverse outcome related to a vaccine or medicine must be systematically included in the differential diagnoses at any healthcare facility [48].

Tackling the fragmentation of existing systems is also crucial. Parallel platforms hinder information flow and can overburden reporters, leading to “reporting fatigue.” A practical solution would be to implement a unified system for collecting minimal data on all critical pregnancy outcomes to provide insights into potential safety concerns. In addition, data on pregnancy outcomes collected from research institutions ought to be shared with the regulators and MoH to enable an end–to–end system that will detect and evaluate safety issues.

The potential of EMR to capture AEFIs in real-time, including those related to maternal vaccines, has been highlighted in previous studies [23, 46, 49, 50]. The EMR systems already implemented by the MoH and research institutions for patient management offer a valuable opportunity to achieve this. The existing UgandaEMR can be enhanced with features that support tracking pregnant women and linking their data to their offspring. This would align with existing workflows within the health facilities. However, two key considerations must be addressed: first, ensuring seamless data access and linkage among all stakeholders involved in maternal vaccine safety monitoring, and secondly, thorough training of healthcare providers on accurate data entry. Even the best EMR system will fail without correct and complete data.

Training health workers at the point-of-care facility to identify, assess, and report both relevant adverse events in pregnant mothers and unexpected effects that may signal safety issues, is critical. Training healthcare professionals to carefully consider current and past treatments in diagnoses will improve the identification of safety issues, including among pregnant women and newborns.

Additionally, consistency in using standardized medical terms is important for clear communication, data analysis and equipping the national vaccine safety committee with the expertise to assess potential causal links between events and adverse pregnancy outcomes. Similar studies in India [51] and Ghana [52] confirm that lack of knowledge and misdiagnosis are the critical barriers to reporting adverse events. Therefore, the PV program should leverage training and standardized case definitions for AEFMI [53].

In cases where vaccines are not registered or indicated for use in pregnant women, there is need for clear policies and communication including safety monitoring. Other studies have emphasized the importance of clear communication about the rationale and recommended course of action between regulatory bodies, public health authorities, and healthcare providers should AEFMI occur and when products are used in emergencies [25, 54].

### Strengths and Limitations

The key strength of this study was the ability to gather insights from informants and group discussions that included both policymakers and implementers. This enabled us to explore real-world experiences and activities that influence the practical implementation of the maternal vaccine safety surveillance. However, the study has some limitations as some stakeholders and geographical regions could have been excluded due to the small sample size, thus limiting the breadth of perspectives. The sampling approach used in the study might limit the generalizability of our findings. These limitations should be considered when interpreting the study results.

## Conclusion and Recommendations

Our study identified gaps in Uganda’s pharmacovigilance system for maternal vaccines that can be addressed. We recommend a multi-pronged approach, including developing coherent policies, strengthening active surveillance through existing systems, and harnessing healthcare provider knowledge and advisory committee capacity in causality assessment. Our findings provide a basis for prioritizing interventions to build an integrated national surveillance system that can effectively monitor both existing and future maternal vaccines in Uganda.

## Supplementary Material

SDC1

SDC2

SDC3

SDC4

SDC5

SDC6

SDC7
