# Supplementary material for: Pharmacovigilance for Vaccines Used in Pregnancy: A Gap Analysis From Uganda
Source: Pediatr Infect Dis J. Author manuscript; Available in PMC 2025 Feb 21. (PMC7617404; doi:10.1097/INF.0000000000004705)
Supplement: SDC1 [file EMS202778-supplement-SDC1.docx]

**SUPPLEMENTAL DIGITAL CONTENT 1.** Study interview guide

**Semi-structured interview guide for KII and FGD**

**Study: Landscape assessment to understand maternal Vaccine pharmacovigilance in Uganda.**

Objectives:

1. Map stakeholders involved in regulation, policy setting, collection and reporting of adverse events following maternal vaccination and their linkages
2. Understand the existing methods, tools, and information flow for collecting and flow of adverse events following maternal vaccination
3. Mapping of current Electronic medical records (EMR) and registries rollout and how they interface with the national drug safety reporting system, especially regarding maternal vaccination

**Introduction instructions for data collectors/interviewers**

A: Explain the study objectives to participants and seek written or verbally recorded informed consent.

B: Record Bio-data:

To record details such as gender, qualification (certificate/diploma/degree), and position in the institution/cadre of participants.

C. Open-ended questions:

1. To start, please tell us about yourself (what you do in this job/role and how long you have been at it)

- The question seeks to find out the role and knowledge in pharmacovigilance, immunisation and Antenatal care

1. What are the vaccines currently administered to pregnant women?
2. How is your healthcare facility involved in safety surveillance in Uganda?

- Probe: How do they engage in PV activities

1. How is your organization involved in monitoring the safety of vaccines administered to pregnant women?

- This is a specific question about reporting side effects of vaccines or medicines used in pregnant women.
- If they have for Pregnant women, let them explain the setup, what they monitor, the data collection tools and databases

1. Where do you get updated safety information about vaccines given to pregnant mothers?

- This seeks to know if they are equipped with knowledge about the safety of vaccines and products given to women when pregnant. Where do they get such information?

**Additional questions for NDA, WHO, MoH-MCH program**

1. How do you disseminate safety information related to pregnant mothers?

Workshop, guidelines, circulars

1. Do you have risk management plans for drugs authorized for use in pregnant women? Probe for details and if possible, get a document describing the risk management plan

D. Data collection

| 1. What tools are you using to collect adverse event reports, e.g., paper forms, hotline, electronic reporting? Electronic medical records, etc.? Are the tools used for reporting AE following immunization in pregnant women |  |
| --- | --- |
| 1. In your opinion, what is the most preferred reporting tool for reporting ADE |  |
| 1. What approach or method do you have to monitor the safety of Vaccine?   Is it a passive system, or do you actively solicit AE? |  |
| 1. What share of reports come through active surveillance versus spontaneous reporting? |  |
| 1. What are the challenges you face in adverse event data collection? |  |
| 1. What improvements would you like to see in adverse event data collection for products used in maternal vaccination? |  |
| \| 1. Could you please share the proportion i.e. the adverse reports recorded out of the total number of pregnant women vaccinated in your institution? \| \| --- \|   E. Data storage and management   \| 1. What system is the system used to store & manage adverse event data, e.g. VigiFlow, DHIS2, electronic medical records? 2. For what purpose are these systems used, e.g. for data collection, data storage, signal detection, transferring data to national pharmacovigilance Centre \| \| --- \| | |
| 1. Which organizations have access to the adverse event data in these systems?   What gaps exist? | |
| 1. For each data collection tool used, how does data flow into the system: | |
| 1. Is it a direct link or manual upload? (This is only asked if applicable) | |
| 1. Who does it, from which organization, at what level (e.g. district, region, national)? | |
| 1. For paper forms, where & how is the data inputted into the electronic system? Are Excel line lists used as an interim step? | |
| 1. What challenges are faced in this data entry process? | |
| 1. With which organizations is adverse event data shared? How and in what format? | |
| 1. What assessment or analysis is done on safety data? | |
| 1. Which organizations & people are involved in this review or analysis? | |
| 1. What is the frequency of this review or analysis? | |
| 1. Is the assessment of causality conducted? If so, how? | |
| 1. Are all cases investigated? If not, which cases are investigated? Who decides this, and how is this determined? | |
| 1. Who does the investigation? What is the timeline for the investigation? | |
| 1. For which cases is causality assessment performed? | |
| 1. What is the timeline for causality assessment? | |
| 1. Which organization or committee is responsible for causality assessment? | |
| 1. How frequently is causality assessment done? | |
| 1. What are the outputs of the causality assessment? | |
| 1. Which organization makes decisions on causality assessment outcomes & liaises with the Pharmaceutical companies ? | |
| 1. How frequently and in what format are these outputs shared with the NRA? | |
| 1. Are these outputs shared with any other organization?   (e.g. MoH) | |

Thank you for your participation.
