# Supplementary material for: Pharmacovigilance for Vaccines Used in Pregnancy: A Gap Analysis From Uganda
Source: Pediatr Infect Dis J. Author manuscript; Available in PMC 2025 Feb 21. (PMC7617404; doi:10.1097/INF.0000000000004705)
Supplement: SDC2 [file EMS202778-supplement-SDC2.docx]

**SUPPLEMENTALDIGITAL CONTENT 2.**  Participant category, structure and interview distribution among respondents.

| **Stakeholders Category** | **Structural Level** | **Location** | **Assessment method** | **Number of participants** | Participant role in the organisation represented |
| --- | --- | --- | --- | --- | --- |
| World Health Organisation (WHO) country office | International/National | Kampala | KII | 1 | Providing technical support and guidance to the Ministry of Health |
| National Regulatory Authority (NRA) | National | NDA, Kampala | KII | 2 | Pharmacovigilance activities |
| Ministry of Health (MOH)-maternal and child health Program | National | MOH, Kampala | KII | 2 | Maternal and childhood health activities |
| Expanded Program on Immunization (EPI) | National  District | MOH,Kampala  District surveillance officer [1 from each sub-region] | KII | 1  3 | Planning for vaccination and AEFI surveillance    Vaccine surveillance, monitoring cold chain system, and vaccine distribution |
| Research/other institutions. | District | Medical Research Council  Baylor  MUJHU  Infection Disease institute | KII | 1  1  1  1 | Managing both investigational and non-investigational products  Support pharmacovigilance systems for referral hospitals and centres of excellence. |
| *Healthcare providers working in ANC | National tertiary level  Regional tertiary care  District tertiary care  District primary care  District/.Research institution | Kawempe NRH -Central region  Mbarara RRH -Western region  Iganga Hospital -Eastern region  Namulonge HCIII – Central region  MUJHU-Central | FGD | 8  8  8  8  7 | ANC services |

KII= Key informant Interview, FGD= Focus group discussion, MUJHU= Makerere University -John Hopkins University Research Collaboration, AEFI=Adverse events following immunization.NRH=National Referral Hospital, RRH=Regional Referral Hospital.

*Healthcare providers involved in the FGD were from facilities selected from three geographical regions of the country (Central. Western, and Eastern)
