# Supplementary material for: Pharmacovigilance for Vaccines Used in Pregnancy: A Gap Analysis From Uganda
Source: Pediatr Infect Dis J. Author manuscript; Available in PMC 2025 Feb 21. (PMC7617404; doi:10.1097/INF.0000000000004705)
Supplement: SDC3 [file EMS202778-supplement-SDC3.docx]

**SUPPLEMENTAL DIGITAL CONTENT 3.** Key study findings and respondents except

| Key findings | Respondent excerpt |
| --- | --- |
| Vaccines given to pregnant women     1. Vaccines other than tetanus approved for use were given during disease outbreaks such as yellow fever, cholera, etc. | *“… Response campaigns such as yellow fever and meningitis A have been conducted for vaccinations that are also given to women. Pregnant women are considered a high-risk group for COVID-19, and thus, they have been included in the COVID-19 vaccine campaigns. Tetanus toxoid is a routinely administered vaccine, while the cholera vaccine is not excluded for women.”* ***(KI_MoH)*** |
| Stakeholders involved in monitoring vaccines   1. Various stakeholders were involved in vaccine safety monitoring at various levels, including the Ministry of Health(MoH), National Drug Authority(NDA), district focal persons, research institutions involved in routine care, etc | *“I would not say we have a special arm dedicated to pregnant women, but generally, we have a focal person for vaccines who works with UNEPI and WHO to monitor vaccines. The multi-sectoral team, UNEPI, WHO, NDA, and Ministry of Health, handles them. Then we have an AEFI expert committee that periodically reviews serious AEFIs.”* ***(KI_NDA****)*  *“One of our mandates is always to update our policy and guidelines based on the emerging situation and the disease burden in the country. So, we also conduct training and supportive supervision programs for districts to ensure that they align with the Ministry policy and other global guidelines.” (****KI_MoH)”***  “*As the surveillance focal person, I normally go during my supervision, and when I go, I mentor health workers on what is expected. After talking, I leave my telephone number behind and also give it to the communities where mothers come from because we have VHTs. So, we tell these mothers that whenever they get something unusual, they report it to the VHT. So, these VHTs sometimes call me and tell me, and in case they go to the health facility where I provided my number, they have to communicate with me.”* ***(KI_District Surveillance focal person)*** |
| The method used for monitoring vaccines used in maternal immunisation   1. There was no system dedicated to maternal vaccine surveillance 2. Passive surveillance mainstay | *“We don’t have a special system for pregnant women. We rely on the surveillance system for all. So, if it can work for the children, it can also work for the pregnant women. So, it’s the same system by the health workers interacting with these women and the same reporting system and management protocols based on how the person presents.”* ***KI_MoH****)*  *“I think, generally, pregnant women have not been on the radar, which I guess is an oversight because they are a special group. So, all my answers on reporting would be general”* ***(KI_NDA)***  *“It is passive. Active is a bit expensive because we have to call and follow the patients. One way is going through the records and calling them. Another way is looking at the patient files. You look at the patient files to see if this could be a potential adverse event because of what happens after the tetanus vaccine. It is very trying because you have a whole workload and many vaccines to cover.”* ***(KI_ 07)***  *“They chose facilities in the country to be “sentinel sites”. That means that when a new drug is introduced in the HIV program, these sites are supposed to be used to collect data to understand how safe the drug is. They are in all the regional referral hospitals.” (****KI_researcher )*** |
| Data collection, storage and information flow   1. Both manual and electronic platforms are available for reporting adverse events and are managed by different stakeholders. 2. There is parallel reporting  - NDA is responsible for ADR reporting forms, toll-free lines, USSD codes, Medsafety App, and WhatsApp - MoH responsible for DHIS, Uganda EMR systems - Research institutions have EMR and register | “…*We use the electronic system to store that data, but there is also a book where we put the data first, and then we report in the electronic system; for data collection, I use the reporting form because we report on that form and then keep an electronic copy. Then the electronic system we usually use them when we are putting them in to be seen by the responsible people…”(* ***KI_08)***  *“…A complaint comes in from the client, the form is filled, and it is registered in the register; we get it from the register and put it into the AEFI form and then from the EFI form, we put it in the electronic system…” (****KI_ 07)***  *“…another thing, sometimes we can get the information and fill it out online, like using the ODK as she is telling you, and we send it directly to the dashboard of the ministry..” (****KI_ 08)***  *“…data collection is by the health worker at the clinic, and then the data entrant will enter from the hard copy to EMR or even sometimes some of us enter directly into EMR and then the data manager is the one who uses the DHIS2…” (****KI 07)***  *“So, in our institution, we use EMR for most of the clinical data. That is the Electronic Medical Record(EMR), so, for all clinical data, clinical management, we use EMR”. (****KI_researcher*** *)*  *“…we have our electronic patient clinic management tool –integrated clinic enterprise application -ICEA, where we complete all the routine clinic data from the visitor, the visit date, the baseline, the social demographic, the drug history, the clinic… each clinic visit. So, it has a section where you can document adverse events, and also, if you are changing the drug, you can put the reason, whether it is toxicity or it is something like that. The system is also used to follow up with baby or pregnant mothers. So, we can document if a mother has had a* bad and a good outcome and what drugs she was or has been on. Maybe the challenge with this system is that we can’t pull so much data on what she might have gotten from outside, what we didn’t give her…” (**KI**  **researcher)**  “*The other thing is that we don’t currently report to the ministry or National Drug Authority on the outcomes of our mothers. We are only reporting to this database. The Antiretroviral Pregnancy Registry…It is a USA-funded database”(***KI_researcher** ) |
| Electronic Medical records(EMR) and registries   1. UgnadaEMR was available in hospitals but focused on HIV management. 2. Research institutions used EMRs and registries for research and did not report to MoH or NDA. | “*The HIV clinic has the EMR, which provides data for all their patients. If an ADR happens on that side, they can capture it because they put it in the patient's file and then update the system…”* ***(KI 07)***  *“…we have our electronic patient clinic management tool –integrated clinic enterprise application -ICEA, where we complete all the routine clinic data from the visitor, the visit date, the baseline, the social demographic, the drug history, the clinic… each clinic visit. So, it has a section where you can document adverse events, and also, if you are changing the drug, you can put the reason, whether it is toxicity or it is something like that. The system is also used to follow up with babies or pregnant mothers. So, we can document if a mother has had a bad and a good outcome and what drugs she was or has been on. Maybe the challenge with this system is that we can’t pull so much data on what she might have gotten from outside, what we didn’t give her…”* ***KI researcher***  *“The other thing is that we don’t currently report to the ministry or National Drug Authority on the outcomes of our mothers. We are only reporting to this database. The Antiretroviral Pregnancy Registry…It is a USA-funded database”(KI_researcher )* |
| Barrier to reporting | Respondent excerpt |
| Logistical challenges   1. Time to report due to heavy workload. Health workers perceive collecting data on adverse events as an additional role beyond their job description, 2. There are no tools specific to maternal vaccines. 3. Logistical challenges of lack of data or phone credit to report and follow up cases | *“The major challenge is having people fill out the form because many feel that it is not their obligation or part of their key outputs or performance indicators in their job. So, you have to convince them to fill out these forms*” [**KI_08]**  “  *time and motivation. First of all, clinicians are not motivated to ask about side effects. Then, documentation and reporting and submission of the report.* *They are so busy, and they want to clear the loads. So, time and motivation from the clinical teams can be an issue.* **[KI_ 07]**  *“They should give us allowances. I know they have tried anyway, but the teams need motivation and airtime; they are sacrificing and going beyond their call of duty to send the reports.”*. **[KI_07**]  *“The website and online platform have its challenges, in that, it requires internet data”.* ***[ KI_06]*** |
| Lack of feedback and action | *“The reporters have no motivation to report. And what causes this is their expectation that ‘when I report today, tomorrow NDA should come and immediately take action’. If I am reporting this, they should come and treat me, or they should pay for my associated medical bills... So, they get frustrated that there is no immediate action.”* [**KI_011]**  “*…We try to report, but no one is following up… So, it is as if we are not reporting anywhere. They should* follow up and give us feedback, whether good or bad, but we are also encouraged to keep reporting any adverse effect*.*” [**FGD_06]** |
| Inadequate capacity and skill to manage the ADR   1. Lack of confidence in dealing with AEFI due to limited information 2. Inadequate capacity of health providers to identify side effects related to vaccines | *“Sometimes, so many patients complain, and you don’t know how best to manage them because they all have the same problem. Then, looking for the solution can also be a challenge.*” [**KI_07**]  “*But in cases where someone is using other herbal concoctions, we might not know. So, we shall just incline on to a vaccine without ruling out the possibility of some other cause*”. [**KI_07**] |
| Inadequate relevant data to enable casualty assessment   1. Incomplete information impeding the ability to undertake causality assessments, | “*Mothers keep on migrating from place to place, from town to villages… so, we end up missing the information on adverse events and follow up is challenging* [**FGD_06]**  **“***There are poor quality reports not very well detailed***” [KI_011]**  “*Sometimes the patient has not moved with drug or information on drugs. How do you know which batch of vaccine or drug she reacted to? Sometimes, you want to find the age and when it happened, but it is missing in the reporting form*” [**KI_ 07]”** |

*ADR=Adverse drug reaction,AEFI=Adverse events following immunization,DHIS=Distric health information system,*

*KI=Key informant, FGD=Focus group discussion, USSD=Unstructured supplementary service data*
