# Supplementary material for: Pharmacovigilance for Vaccines Used in Pregnancy: A Gap Analysis From Uganda
Source: Pediatr Infect Dis J. Author manuscript; Available in PMC 2025 Feb 21. (PMC7617404; doi:10.1097/INF.0000000000004705)
Supplement: SDC4 [file EMS202778-supplement-SDC4.docx]

**SUPPLEMENTAL DIGITAL CONTENT 4.** Key stakeholders involved in pharmacovigilance in Uganda and their different roles.

| Structural level | Stakeholder | Roles |
| --- | --- | --- |
| National level | **National Drug Authority(NDA)** | - Overall regulatory oversight over drugs and vaccines - Has mandate to coordinate pharmacovigilance and hosts the national pharmacovigilance Centre - Develop and disseminate reporting tools and training of health professionals - Receive information on suspected adverse drug events /adverse following immunization (AEFI) from health facilities - Investigation of serious adverse events and provides the necessary feedback to relevant stakeholders - Signal management activities - Manages the Vigiflow database where information on Adverse Drug Events [ADE] is stored - Publishes quarterly bulletins on the status of the vaccines and drugs that are used in Immunization and vaccination |
|  | **Ministry of Health, including EPI and MCH** | - Sets policy and implementation direction for medicines and vaccines, including implementation of pharmacovigilance across healthcare facilities in the country - Causality assessments for serious AEFIs through the national AEFI committee - Provides support supervision for districts to ensure that they are in line with the relevant policies - Conducts disease surveillance - Investigation of serious AEFI in collaboration with NDA |
|  | **World Health Organization (Country office)** | - Technical support and guidance to the Ministry of Health. Provide related supporting activities in routine immunization, new vaccine introductions, immunization campaigns - Secretariat with EPI and NDA to the National AEFI Committee |
| Regional/district level | **District EPI focal person** | - Oversee monitoring activities related to vaccine pharmacovigilance and cold chain - Investigates serious adverse events following Immunization |
|  | **Health facilities** | - Implement policies and provide healthcare - Reporting of adverse events occurring in patients |
| Others | **Research institutions**  **Implementing partners** | - Conduct safety studies - Supports pharmacovigilance for products used in routine care and administered to study participants - Provide related technical supporting activities in vaccination programs at different levels |

*EPI= Expanded program on immunization, MCH= Maternal and child health*
