# Supplementary material for: Pharmacovigilance for Vaccines Used in Pregnancy: A Gap Analysis From Uganda
Source: Pediatr Infect Dis J. Author manuscript; Available in PMC 2025 Feb 21. (PMC7617404; doi:10.1097/INF.0000000000004705)

**SUPPLEMENTAL DIGITAL CONTENT 5.** Key stakeholders involved in pharmacovigilance of vaccines and flow of adverse event reporting


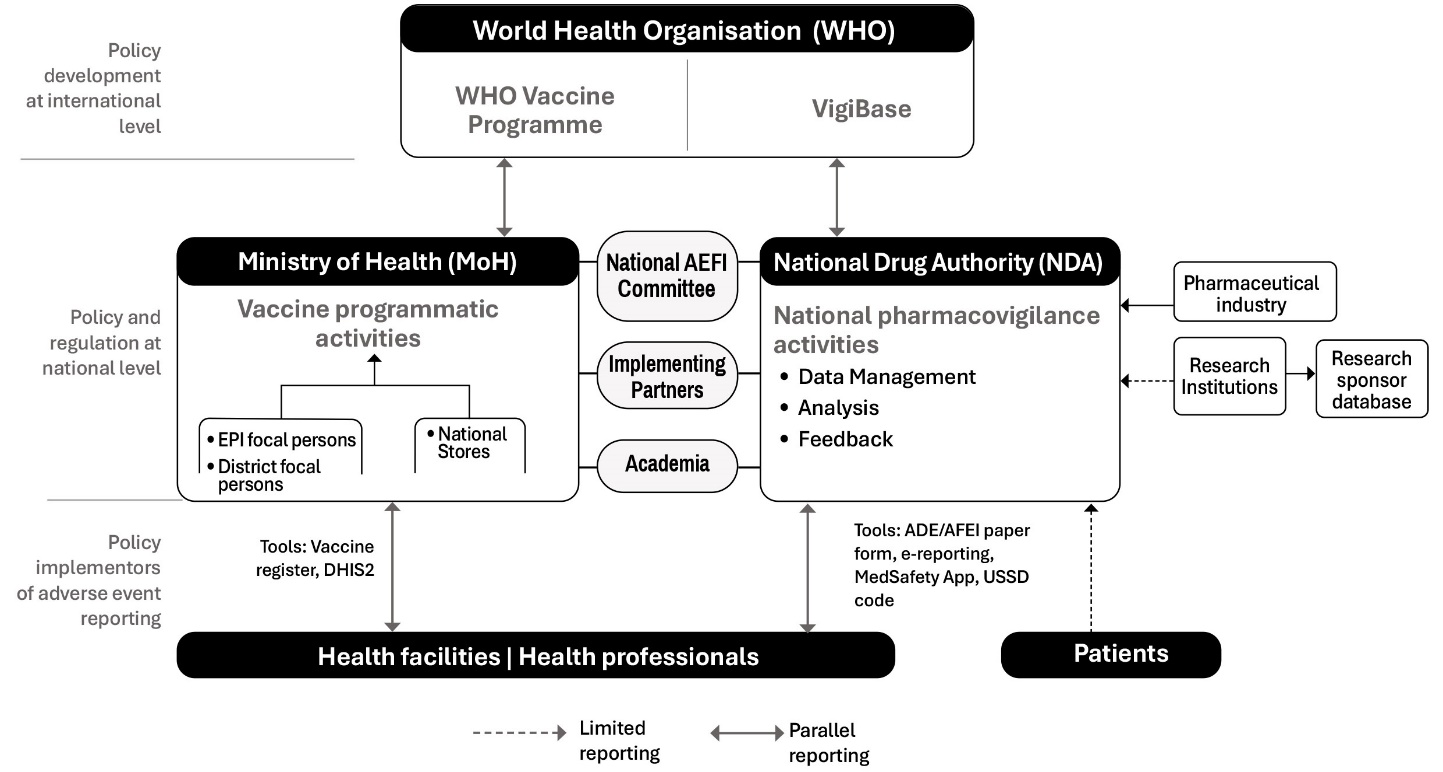

Supplement: SDC5 [file EMS202778-supplement-SDC5.docx]
