# Supplementary material for: Pharmacovigilance for Vaccines Used in Pregnancy: A Gap Analysis From Uganda
Source: Pediatr Infect Dis J. Author manuscript; Available in PMC 2025 Feb 21. (PMC7617404; doi:10.1097/INF.0000000000004705)
Supplement: SDC6 [file EMS202778-supplement-SDC6.docx]

**SUPPLEMENTAL DIGITAL CONTENT 6.** Barriers to reporting adverse events by healthcare providers in Uganda.

| Identified challenges | Respondent excerpt |
| --- | --- |
| Logistical challenges   1. Time to report due to heavy workload. Health workers perceive collecting data on adverse events as an additional role beyond their job description, 2. There are no tools specific to maternal vaccines. 3. Logistical challenges of lack of internet or phone credit to report and follow up cases | *“The major challenge is having people fill out the form because many feel that it is not their obligation or part of their key outputs or performance indicators in their job. So, you have to convince them to fill out these forms*” [**KI_08]**  *“Time and motivation. First, clinicians are not motivated to ask about side effects. Then, documentation and reporting and submission of the report.* *They are so busy, and they want to clear the loads. Therefore, time and motivation from the clinical teams can be an issue.* **[KI_ 07]**  *“They should give us allowances. I know they have tried anyway, but the teams need motivation and airtime; they are sacrificing and going beyond their call of duty to send the reports.”* **[KI_07**]  *“The website and online platform have its challenges, in that it requires internet.”* ***[ KI_06]*** |
| Lack of feedback and action | *“The reporters have no motivation to report. And what causes this is their expectation that ‘when I report today, tomorrow NDA should come and immediately take action’. If I am reporting this, they should come and treat me, or they should pay for my associated medical bills... So, they get frustrated that there is no immediate action.”* [**KI_011]**  “*…We try to report, but no one is following up… So, it is as if we are not reporting anywhere. They should* follow up and give us feedback, whether good or bad, but we are also encouraged to keep reporting any adverse effect*.*” [**FGD_6]** |
| Inadequate capacity and skill to manage the ADR   1. Lack of confidence in dealing with AEFI due to limited information 2. Inadequate capacity of health providers to identify side effects related to vaccines in | *“Sometimes, so many patients complain, and you don’t know how best to manage them because they all have the same problem. Then, looking for the solution can also be a challenge.*” [**KI_07**]  “*But in cases where someone is using other herbal concoctions, we might not know. So, we shall just incline on to a vaccine without ruling out the possibility of some other cause.*” [**KI_07**] |
| Inadequate relevant data to enable casualty assessment   1. Incomplete information impeding the ability to undertake causality assessments, | “*Mothers keep on migrating from place to place, from town to villages… so, we end up missing the information on adverse events and follow up is challenging.”* [**FGD_06]**  **“***There are poor quality reports not very well detailed.***” [KI_011]**  “*Sometimes the patient has not moved with drug or information on drugs. How do you know which batch of vaccine or drug she reacted to? Sometimes, you want to find the age and when it happened, but it is missing in the reporting form.*” [**KI_ 07]”** |

*KI=Key informant, FGD=Focus group discussion*
