# Supplementary material for: Pharmacovigilance for Vaccines Used in Pregnancy: A Gap Analysis From Uganda
Source: Pediatr Infect Dis J. Author manuscript; Available in PMC 2025 Feb 21. (PMC7617404; doi:10.1097/INF.0000000000004705)
Supplement: SDC7 [file EMS202778-supplement-SDC7.pptx]

## Slide 1
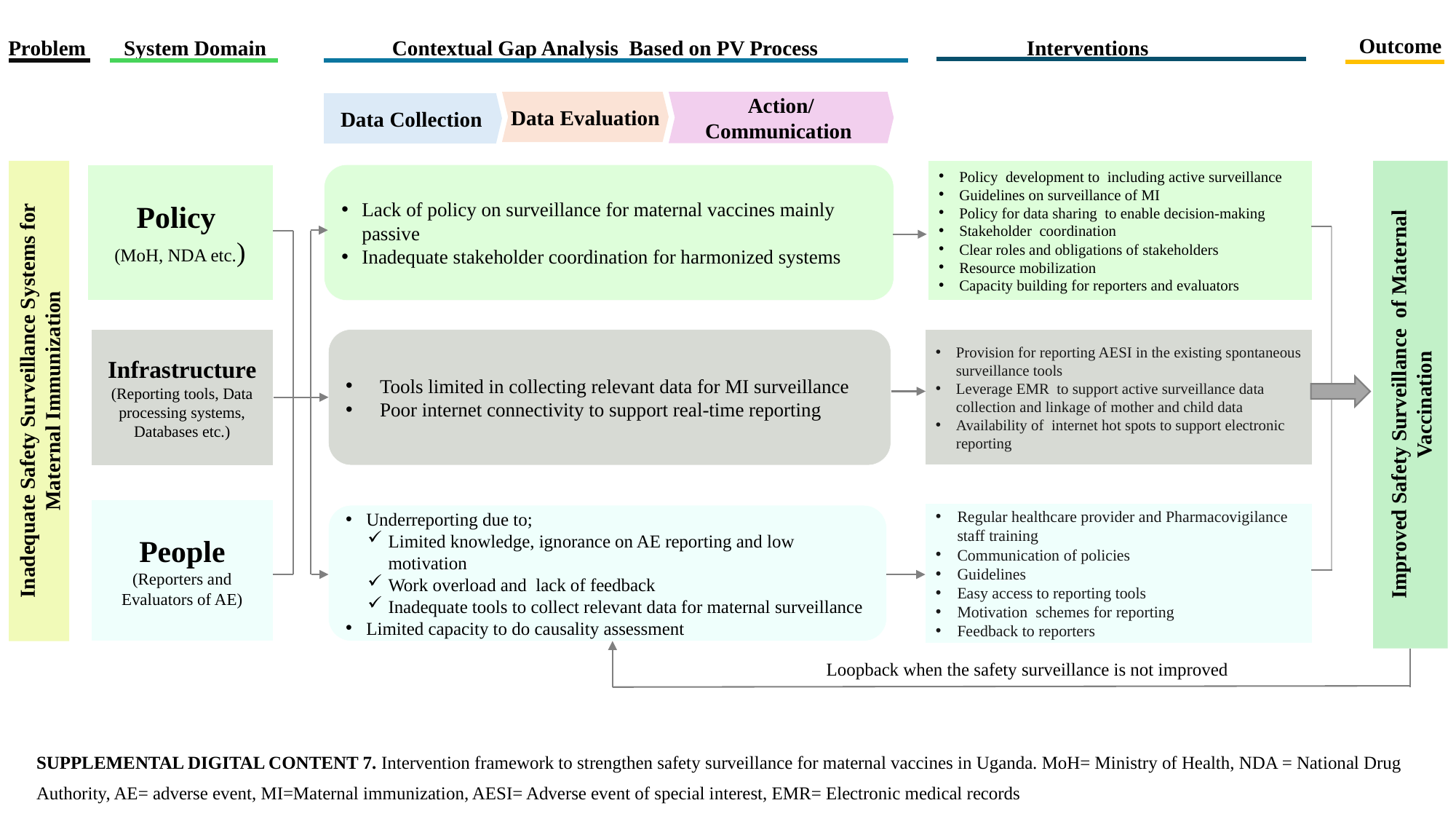

Outcome
Problem
System Domain
Interventions
Contextual Gap Analysis Based on PV Process
Data Evaluation
Action/Communication
Data Collection
Policy development to including active surveillance
Guidelines on surveillance of MI
Policy for data sharing to enable decision-making
Stakeholder coordination
Clear roles and obligations of stakeholders
Resource mobilization
Capacity building for reporters and evaluators
Policy
(MoH, NDA etc.)
Lack of policy on surveillance for maternal vaccines mainly passive
Inadequate stakeholder coordination for harmonized systems
Provision for reporting AESI in the existing spontaneous surveillance tools
Leverage EMR to support active surveillance data collection and linkage of mother and child data
Availability of internet hot spots to support electronic reporting
Infrastructure
(Reporting tools, Data processing systems, Databases etc.)
Tools limited in collecting relevant data for MI surveillance
Poor internet connectivity to support real-time reporting
Improved Safety Surveillance of Maternal Vaccination
Inadequate Safety Surveillance Systems for Maternal Immunization
People
(Reporters and Evaluators of AE)
Regular healthcare provider and Pharmacovigilance staff training
Communication of policies
Guidelines
Easy access to reporting tools
Motivation schemes for reporting
Feedback to reporters
Underreporting due to;
Limited knowledge, ignorance on AE reporting and low motivation
Work overload and lack of feedback
Inadequate tools to collect relevant data for maternal surveillance
Limited capacity to do causality assessment
Loopback when the safety surveillance is not improved
SUPPLEMENTAL DIGITAL CONTENT 7. Intervention framework to strengthen safety surveillance for maternal vaccines in Uganda. MoH= Ministry of Health, NDA = National Drug Authority, AE= adverse event, MI=Maternal immunization, AESI= Adverse event of special interest, EMR= Electronic medical records
